# PIN1 gene variants in Alzheimer's disease

**DOI:** 10.1186/1471-2350-10-115

**Published:** 2009-11-12

**Authors:** Aleksandra Maruszak, Krzysztof Safranow, Katarzyna Gustaw, Beata Kijanowska-Haładyna, Katarzyna Jakubowska, Maria Olszewska, Maria Styczyńska, Mariusz Berdyński, Andrzej Tysarowski, Dariusz Chlubek, Janusz Siedlecki, Maria Barcikowska, Cezary Żekanowski

**Affiliations:** 1 Department of Neurodegenerative Disorders, Mossakowski Medical Research Centre, Polish Academy of Sciences, Pawińskiego 5, 02-106 Warszawa, Poland; 2 Department of Biochemistry and Medical Chemistry, Pomeranian Medical University, Al. Powstańców Wielkopolskich 72, 70-111 Szczecin, Poland; 3 Alzheimer's Research Unit, Institute of Agricultural Medicine, Jarczewskiego 2, 20-950 Lublin, Poland; 4 Psychogeriatric Department, Institute of Psychiatry and Neurology, Sobieskiego 9, 02-957 Warszawa, Poland; 5 Department of Molecular Biology, Maria Skłodowska-Curie Memorial Cancer, Center and Institute of Oncology, W.K. Roentgena 5, 02-781 Warszawa, Poland; 6 Neurology Clinic, Warsaw Central Clinic Hospital CSK MSWiA, Warszawa, Poland

## Abstract

**Background:**

Peptidyl-prolyl isomerase, NIMA-interacting 1 (PIN1) plays a significant role in the brain and is implicated in numerous cellular processes related to Alzheimer's disease (AD) and other neurodegenerative conditions. There are confounding results concerning PIN1 activity in AD brains. Also *PIN1 *genetic variation was inconsistently associated with AD risk.

**Methods:**

We performed analysis of coding and promoter regions of *PIN1 *in early- and late-onset AD and frontotemporal dementia (FTD) patients in comparison with healthy controls.

**Results:**

Analysis of eighteen *PIN1 *common polymorphisms and their haplotypes in EOAD, LOAD and FTD individuals in comparison with the control group did not reveal their contribution to disease risk.

In six unrelated familial AD patients four novel *PIN1 *sequence variants were detected. c.58+64C>T substitution that was identified in three patients, was located in an alternative exon. *In silico *analysis suggested that this variant highly increases a potential affinity for a splicing factor and introduces two intronic splicing enhancers. In the peripheral leukocytes of one living patient carrying the variant, a 2.82 fold decrease in *PIN1 *expression was observed.

**Conclusion:**

Our data does not support the role of *PIN1 *common polymorphisms as AD risk factor. However, we suggest that the identified rare sequence variants could be directly connected with AD pathology, influencing *PIN1 *splicing and/or expression.

## Background

PIN1 is a ubiquitously expressed protein, belonging to the evolutionarily conserved peptidyl-prolyl isomerase (PPIase) family. PIN1 isomerizes p(Ser/Thr)-Pro motifs in the target proteins, which leads to the alteration of their structure, function, intracellular localization and/or stability [1]. Previous studies have demonstrated that PIN1 plays a crucial role in multiple cellular processes and, likewise, it has been implicated in pathogenesis of several diseases, including cancer, inflammation to neurodegenerative diseases [2-8].

The gene encoding PIN1 maps to chromosome 19p13.2, a region associated with late-onset Alzheimer's disease (LOAD) [9]. Moreover, *PIN1 *is the only known gene whose knockout in mice can cause both Tau and Aβ-related pathologies in an age-dependent manner [4,10]. It was shown that PIN1-catalysed conformation change of pT668 could prevent amyloidogenic processing of APP [10]. Additionally, in a similar manner PIN1 may indirectly reverse the hyperphosphorylation of Tau, restoring its ability to bind microtubules, as well as inhibit GSK3β phosphorylation [5,11]. As overexpression of PIN1 in vitro induced a reduction in amyloidogenic processing of APP, it has been proposed that functional PIN1 could prevent or slow down AD onset [10]. On the other hand, PIN1 dysfunction or down-regulation e.g. under the oxidative stress, would favor *cis *form of pT668 APP and toxic Aβ production, leading finally to neurodegeneration [5,12,13]. However, there are confounding results considering the activity and the role of PIN1 in AD [14]. PIN1 protein was depleted in hippocampi of AD patients [2,15]. However, others showed that in the cortex of the frontal lobes of MCI and AD patients PIN1 levels and activity were increased compared to healthy controls [16].

Recently, PIN1 expression has been shown to increase during neuronal differentiation, which led to suggestion that PIN1 dysfunction or downregulation could favor cell cycle re-entry [17-19]. This could result in aneuploidy observed in AD patients brains [20]. Indeed, several lines of evidence indicate that disturbed maintenance and segregation of chromosomes, DNA damage and impaired repair could contribute to AD [21-23].

PIN1 downregulation or dysfunction could result not only from oxidative stress, but also could be connected with genetic variability [10,12,15,17,24]. Segat et al. demonstrated that the carriers of PIN1 -842C allele and/or -842C/-667C haplotype have an increased risk of AD, lower age of onset, and reduced PIN1 levels in peripheral mononuclear cells [12]. Moreover, individuals with amnestic MCI recruited from the same population showed a similar genotype distribution of -842 SNP as AD patients in Segat et al. (2007) study [12,25]. However, other studies on the role of *PIN1 *genetic variants in AD did not repeat the initial findings [26-28].

To our knowledge, a thorough analysis of haplotypes that are formed by a set of *PIN1 *SNPs has not been described yet. Moreover, there were no studies on the involvement of *PIN1 *variants in early onset AD (EOAD), familial AD, and FTD, despite the fact that decreased PIN1 expression and depletion of neuronal nuclear PIN1 has been suggested to be a common feature in AD and FTD [7]. Given supporting evidence for PIN1 role in the brain, and yet unresolved influence of *PIN1 *sequence variation in AD and FTD, we decided to perform an exhaustive analysis of *PIN1 *in a group of Polish AD and FTD patients.

## Methods

111 late onset (mean age of onset ± SD: 73.2 ± 5.0 years, range 66-88; 69.4% females) and 49 early onset AD patients (mean age of onset ± SD: 52.6 ± 9.8 years; 57.1% females), and 57 frontotemporal dementia (FTD) patients (mean age of onset: 59.3 ± 12.3 years; 43.9% females) were recruited for the study. Twenty-six patients in the EOAD group have a family history of AD recorded, and 70 patients in the LOAD group claim a family history of dementia. The control group consisted of 104 healthy, non-demented individuals (mean age ± SD: 75.1 ± 5.2 years, range: 68-90; 71.15% females). AD diagnosis fulfilled the criteria of National Institute of Neurological and Communicative Disorders and Stroke - Alzheimer's Disease and Related Disorders Association (NINCDS-ADRDA) for probable AD, whereas FTD was diagnosed according to Lund and Manchester Groups (1994) and Neary (1998) criteria [29,30]. The control subjects had normal mental status test scores and no clinical evidence of cognitive deficits in neurological examination.

All participants or their relatives provided written, informed consent and the study was approved by the Ethics Committee of the MSWiA Hospital in Warsaw in accordance with the principles of the Helsinki Declaration.

*PIN1 *promoter (1545 kb upstream the ATG translation initiation codon; NCBI GenBank AF501321) and coding regions (4 exons with flanking intronic regions of about 100 nt; NCBI GenBank NM_006221, NC_000019.8) were amplified (primers are listed in Additional file 1). Analyzed fragments covered 18 annotated DNA variations (rs7247933, rs4804459, rs2233678, rs35794537, rs2233679, rs2233680, rs7250788, rs35973416, rs28589723, rs2233681, rs2233682, rs2233683, rs2010457, rs11540415, rs34412035, rs11540414, rs3178950, rs35575918).

APOE genotypes were determined as described previously [31]. Patients and controls were stratified into two subgroups, according to *APOE *status: those carrying at least one *APOE4 *allele (*APOE4*+) and *APOE4 *non-carriers (*APOE4*-).

First, sequencing of *PIN1 *in DNA samples from EOAD and FTD patients was performed. Four identified variants in EOAD patients were screened in the control group and LOAD patients using denaturing high performance liquid chromatography (dHPLC). DHPLC analysis was performed as described previously (for DHPLC temperature for the analysis of *PIN1 *gene fragments see Additional file 2) [32,33]. Both groups were also screened for SNPs heterozygous in the Polish population (rs4804459, rs2233678, rs2233679, rs2233682, rs2233683, rs2010457).

The identified promoter DNA substitution was examined for introducing potential differences in transcription factor binding sites using MatInspector (Geneomatix software, Germany, [34]) and Mapper http://bio.chip.org/mapper. The possible effect of identified DNA variants on splicing was investigated using ESEfinder (release 3.0) [35,36] and Automated Splice Site Analyses [37,38]. ESEfinder identifies putative exon splicing enhancers responsive to the human serine/arginine-rich (SR) proteins, whereas Automated Splice Site Analyses evaluates changes in splice site strength based on information theory based models. Analysis of intronic sequences containing mutations were done also with application of RegRNA: A Regulatory RNA Motifs and Elements Finder http://regrna.mbc.nctu.edu.tw/index.html. ConSeq http://conseq.bioinfo.tau.ac.il/ program was used to evaluate the degree of conservation of mutated residue in the protein coding sequence.

Total RNA was isolated from leukocytes of the patient with c.58+64C>T variant and four healthy individuals, using standard TRI Reagent^® ^method, according to manufacturer's procedure (Ambion). The SuperScript First-Strand Synthesis System for RT-PCR (Invitrogen) was used to synthesize first-strand cDNA, using oligo-dT primers and equal amount of RNA from the samples. Then all samples were adjusted to 20 ng/μl cDNA. qPCR was performed in triplicates in a 25 μl reaction mix with 3 μl of diluted cDNA template, using standard SYBR Green protocol on ABI 7500 Sequence Detection System (Applied Biosystems). The thermal cycling conditions comprised an initial denaturation step at 95°C, then 40 cycles of 95°C for 15 s and 60°C for 1 min. The temperature range used for the melting curve generation was from 60°C to 95°C. The dissociation plots indicated a single peak in all reactions.

The primer set for *PIN1 *was designed to span intron 3 in order to distinguish amplified cDNA from genomic DNA. No primer dimers were observed. The level of *PIN1 *mRNA was normalized to that of succinate dehydrogenase complex subunit A (SDHA). The relative quantification method was applied to analyze real-time PCR results. Similar efficiencies of target and reference genes allowed us to use the comparative Ct method (2^-deltadeltaCt^) to calculate relative expression of *PIN1 *in our patient in comparison with four healthy individuals.

Comparisons of allele and genotype frequencies between the affected and the control group were carried out using the chi-square or Fisher exact (2-tailed) tests. The Hardy-Weinberg equilibrium was tested using a chi-square goodness-of-fit test. Kruskal-Wallis test was used to compare age at onset of LOAD symptoms between genotype groups. Statistical difference was accepted at *p *< 0.05.

Haplotype assignment and linkage disequilibrium (LD) between each pair-wise combination of SNPs heterozygous in the studied groups as expressed by D' was calculated using the Haploview 4.0 (http://www.broad.mit.edu/mpg/haploview/, [39]). Haplotypes were inferred using EM algorithm, which was implemented in Haploview [40].

## Results

**Figure 1:**
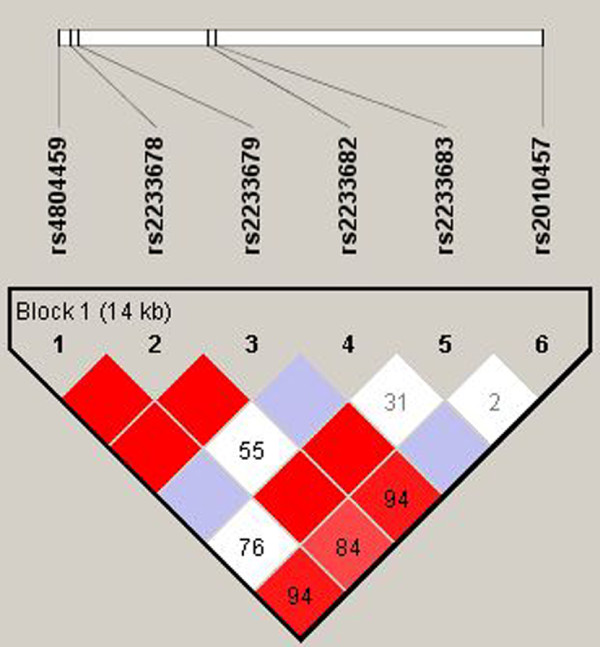
**Pairwise linkage disequilibrium between the six genotyped SNPs in a 14 kb region of *PIN1 *(Haploview 4.1) for combined group (n = 321) of all patients (LOAD, EOAD and FTD) and controls**. Numbers inside the squares represent the D' value expressed as a percent. Squares without numbers represent D' values of 1.0, indicative of complete linkage disequilibrium. Darker-shaded squares represent pairs with LOD score for linkage disequilibrium of = 2, light grey squares represent D' = 1 but LOD <2, and white squares represent LOD <2 and D' <1.0.

Genotyping promoter region and 4 exons with adjacent flanking intron sequences of the *PIN1 *gene revealed six heterozygous polymorphisms (rs4804459, rs2233678, rs2233679, rs2233682, rs2233683, rs2010457) that were included in the further analysis. SNPs rs4804459, rs2233678, rs2233679 and rs2010457 were in linkage disequilibrium in LOAD, EOAD, FTD and control groups (Figure 1). SNPs rs2233682 and rs2233683 had low minor allele frequency.

Genotype and allele frequencies of investigated polymorphisms were similar in LOAD, EOAD, FTD patients in comparison with the controls. Distribution of polymorphisms heterozygous in the studied groups is presented in Table 1. All observed genotype frequencies in the affected and the control group were in the Hardy-Weinberg equilibrium (p > 0.05). The statistical power for comparison of the LOAD (n = 111) and the control (n = 104) groups was sufficient to detect with 80% probability true differences of the allele and haplotype frequencies in the range from 5% (for the most rare alleles) to 12% (for the most common alleles). The respective detectable differences were 6% to 17% for both EOAD (n = 49) and FTD (n = 57) groups when compared to the controls.

**Table 1:** Frequencies of six heterozygous in the Polish population PIN1 polymorphisms

SNP	Genotype n (%)				Allele n (%)		
			
rs4804459	GG	GC	CC	*p*-value	G	C	*p*-value
Controls	3 (2.88)	33 (31.73)	68 (65.38)	controls vs LOAD patients: p = 0.925	39 (18.75)	169 (81.25)	controls vs LOAD patients: p = 0.841
			
LOAD patients	2 (1.80)	36 (32.43)	73 (65.77)		40 (18.02)	182 (81.98)	

EOAD patients	3 (6.12)	13 (26.53)	33 (67.35)	controls vs EOAD patients p = 0.504	19 (19.4)	79 (80.6)	controls vs EOAD patients: p = 0.887

FTD patients	1 (1.75)	22 (38.60)	34 (59.65)	controls vs FTD patients: p = 0.462	24 (21.05)	90 (78.95)	controls vs FTD patients: p = 0.617

				Differences between 4 groups: p = 0.668			Differences between 4 groups: p = 0.925

							
**rs2233678**	**GG**	**GC**	**CC**	***p*-value**	**G**	**C**	***p*-value**

Controls	82 (78.85)	21 (20.19)	1 (0.96)	controls vs LOAD patients: p = 0.715	185 (88.94)	23 (11.06)	controls vs LOAD patients: p = 0.439
							
LOAD patients	83 (74.77)	26 (23.42)	2 (1.80)		192 (86.48)	30 (13.51)	

EOAD patients	34 (69.39)	15 (30.61)	0 (0.00)	controls vs EOAD patients: p = 0.323	83 (84.7%)	15 (15.3)	controls vs EOAD patients: p = 0.263

FTD patients	46 (80.70)	10 (17.54)	1 (1.75)	controls vs FTD patients: p = 0.924	102 (89.47)	12 (10.53)	controls vs FTD patients: p = 0.885

				Differences between 4 groups: p = 0.673			Differences between 4 groups: p = 0.632

							

**rs2233679**	**CC**	**CT**	**TT**	***p*-value**	**C**	**T**	***p*-value**

Controls	9 (8.65)	45 (43.27)	50 (48.08)	controls vs LOAD patients: p = 0.928	63 (30.3)	145 (69.7)	controls vs LOAD patients: p = 0.708
							
LOAD patients	11 (9.91)	49 (44.14)	51 (45.95)		71 (31.98)	151 (68.02)	

EOAD patients	5 (10.20)	25 (51.02)	19 (38.78)	controls vs EOAD patients: p = 0.560	35 (35.7)	63 (64.3)	controls vs EOAD patients: p = 0.343

FTD patients	3 (5.26)	30 (52.63)	24 (42.11)	controls vs FTD patients: p = 0.460	36 (31.58)	78 (68.42)	controls vs FTD patients: p = 0.806

				Differences between 4 groups: p = 0.821			Differences between 4 groups: p = 0.823

							

**rs2233682**	**GG**	**GA**	**AA**	***p*-value**	**G**	**A**	***p*-value**

Controls	103 (99.04)	1 (0.96)	0 (0.00)	controls vs LOAD patients: p = 1.00	207 (99.52)	1 (0.48)	controls vs LOAD patients: p = 1.00
							
LOAD patients	110 (99.10)	1 (0.90)	0 (0.00)		221 (99.55)	1 (0.45)	

EOAD patients	48 (97.96)	1 (2.04)	0 (0.00)	controls vs EOAD patients: p = 1.00	97 (98.98)	1 (1.02)	controls vs EOAD patients: p = 1.00

FTD patients	57 (100.00)	0 (0.00)	0 (0.00)	controls vs FTD patients: p = 1.00	114 (100.00)	0 (0.00)	controls vs FTD patients: p = 1.00

				Differences between 4 groups: p = 0.654			Differences between 4 groups: p = 0.655

							

**rs2233683**	**CC**	**CT**	**TT**	***p*-value**	**C**	**T**	***p*-value**

Controls	100 (96.15)	4 (3.85)	0 (0.00)	controls vs LOAD patients: p = 0.540	204 (98.1)	4 (1.9)	controls vs LOAD patients: p = 0.617
			
LOAD patients	104 (93.69)	7 (6.31)	0 (0.00)		215 (96.85)	7 (3.15)	

EOAD patients	49 (100.00)	0 (0.00)	0 (0.00)	controls vs EOAD patients: p = 0.306	98 (100.00)	0 (0.00)	controls vs EOAD patients: p = 0.310

FTD patients	57 (100.00)	0 (0.00)	0 (0.00)	controls vs FTD patients: p = 0.298	114 (100.00)	0 (0.00)	controls vs FTD patients: p = 0.301

				Differences between 4 groups: p = 0.089			Differences between 4 groups: p = 0.095

							

**rs2010457**	**AA**	**AG**	**GG**	***p*-value**	**A**	**G**	***p*-value**

Controls	52 (50.00)	45 (43.27)	7 (6.73)	controls vs LOAD patients: p = 0.923	149 (71.6)	59 (28.4)	controls vs LOAD patients: p = 0.752
			
LOAD patients	54 (48.65)	48 (43.24)	9 (8.11)		156 (70.3)	66 (29.7)	

EOAD patients	20 (40.82)	25 (51.02)	4 (8.16)	controls vs EOAD patients: p = 0.484	65 (66.3)	33 (33.7)	controls vs EOAD patients: p = 0.322

FTD patients	24 (42.11)	28 (49.12)	5 (8.77)	controls vs FTD patients: p = 0.504	76 (66.7)	38 (33.3)	controls vs FTD patients: p = 0.264

				Differences between 4 groups: p = 0.926			Differences between 4 groups: p = 0.703

There were significantly more *APOE4 *carriers among the LOAD patients (63.06%) in comparison to the control group (21.15%) (χ^2 ^= 38.52, df = 1, p < 0.00001). Stratification of the LOAD patients and the control group according to the *APOE4 *status had no influence on their allele or genotype distribution - there were no significant differences between the groups (p > 0.1, data not shown). In addition, none of the studied polymorphisms affected the age at onset of LOAD symptoms (p > 0.1, data not shown).

Polymorphisms heterozygous in the studied groups determined 3 major haplotypes (Table 2). Among them, the most frequent one, CGTGCA was present in >64% chromosomes. GGCGCG and CCCGCG had a frequency >17% and >9%, respectively. Other identified haplotypes had minor frequencies (<3%). None of the haplotypes was associated with disease status (Table 2).

**Table 2:** Frequency of *PIN1 *haplotypes in LOAD, EOAD and FTD patients compared with the control group

Haplotype *	Controls (%)	All patients (LOAD, EOAD and FTD) together (%)	p value for all patients together vs controls	LOAD (%)	p value for LOAD vs controls	EOAD (%)	p value for EOAD vs controls	FTD (%)	p value for FTD vs controls
CGTGCA	68.7	66.1	0.510	67.1	0.717	64.3	0.438	65.8	0.588
GGCGCG	17.8	18.4	0.844	17.6	0.951	18.4	0.902	20.2	0.599
CCCGCG	8.7	11.3	0.307	9.9	0.654	15.3	0.080	10.5	0.581
CGTGCG	1.0	1.2	0.835	0.9	0.948	0.0	0.333	2.7	0.253
CCCGTA	1.0	1.2	0.838	2.3	0.294	0.0	0.329	0.0	0.291
GGCGCA	1.0	0.5	0.458	0.0	0.149	1.0	0.962	0.9	0.942
CCCGTG	0.9	0.5	0.459	0.9	0.954	0.0	0.331	0.0	0.296
CGCACA	0.5	0.5	0.972	0.5	0.963	1.0	0.585	0.0	0.458
CCCGCA	0.5	0.0	0.151	0.0	0.303	0.0	0.495	0.0	0.463
CGCGCA	0.0	0.2	0.488	0.5	0.332	0.0	-	0.0	-
GCCGCG	0.0	0.2	0.488	0.5	0.332	0.0	-	0.0	-

* The five haplotype loci correspond to SNPs 1-6 in Figure 1

The number of haplotypes within the 14 kb block was greater than the number of SNPs plus one. However, the excess of haplotypes (11 vs 6+1, respectively, Table 2) was still small, which could suggest relatively few recombination events in the past.

Four novel *PIN1 *variants were identified in six unrelated patients with familial AD (summarized in Table 3). All of identified variants were nucleotide substitutions absent in the control group. We detected one promoter mutation (g.9805834T>C, GenBank AF501321), localized 1187 bases upstream the translation start codon. In exon 1 a silent substitution was found (c.24C>T, GenBank NM_006221 and NC_000019.8), changing the third base of codon 8. Two other variants were localized in introns, c.58+64C>T in intron 1 and c.382+105C>T in intron 3. No sequence alterations were detected in the patients with FTD. As PIN1 was postulated to play an important role in oncogenesis, any recognized tumor or cancer in life history of analyzed patients with *PIN1 *variants was indicated in Table 3.

**Table 3:** New sequence variants in *PIN1 *and brief description of their carriers.

Variant	Diagnosis	Gender	Age at onset (years)	family history of dementia (+/-)	APOE genotype	Tumors
**g.9805834T>C**	LOAD	male	70	+ (mother had memory and behavioral disturbances at the age of 60)	34	benign prostate hyperplasia
**c.24G>T**	EOAD	female	52	+ (mother and father's mother)	33	no data
**c.58+64C>T**	EOAD	female	51	+ (father)	44	goiter, benign thyroid tumor
	LOAD	female	71	+ (mothers's brother suffered from dementia)	34	osteoma ossis frontalis
	LOAD	female	70	+ (father in his eighties developed AD symptoms, sister at the age of 66 had psychiatric problems)	34	nodular goitre, breast cancer
**c.382+105C>T**	EOAD	male	48	+ (father had dementia and died at the age of 73)	34	none

Unfortunately, out of the six mutation carriers, only one was available for further investigation. The other five individuals died before the conduction of the study. As Fanghänel et al. (2006) suggested, Pin1 activity is mainly controlled by its expression level. Therefore, in order to asses *PIN1 *expression in the carrier of variant c.58+64C>T, we performed quantitative PCR. Relative quantification, using endogenous control gene, SDHA, and four healthy individuals for data normalization, revealed a 2.82 decreased *PIN1 *mRNA level in the patient (Additional file 3).

## Discussion

Several lines of evidence indicate the importance of PIN1 in the nervous system. PIN1 is expressed in different brain regions at least three-fold higher than in other tissues [41] and has been postulated to be involved in neuronal differentiation and in maintaining normal neuronal functions and their postmitotic state [3,4]. Moreover, PIN1 was demonstrated to have a pivotal role in protecting against age-related neurodegeneration [4]. In Alzheimer's disease PIN1 depletion was related to exacerbated tau hyperphosphorylation, generation of NFT and neurotoxic Aβ and subsequent amyloid plaque formation. Additionally, PIN1 depletion was suggested to contribute to neuronal apoptosis [42].

To test the hypothesis that PIN1 dysfunction in AD and/or FTD could be connected with genetic variability, we analyzed the promoter and coding regions of *PIN1*.

Our data does not support the role of 18 common *PIN1 *polymorphisms as AD or FTD risk factors. Neither individual alleles, nor haplotypes were associated with EOAD, LOAD or FTD risk. Our findings conflicted with Segat et al. (2007) reports of -842C SNP and -842C/-667C haplotype association with AD in the Northern Italian population, however, they were consistent with the data from the American, French and other Italian cohorts [26-28]. The identified variants g.9805834T>C, c.24C>T and c.382+105C>T are all located on the most prevalent haplotypes, CGTGCA/GGCGCG. The patients with variant c.58+64C>T belonged to diplotypes CGTGCA/CGCGCA, CCCGCG/CGCACA and CCCGTA/CGCACA.

We did not identify sequence variants or haplotype associated with the risk for FTD. On one hand enrolling FTD patients without neuropathological characterization could be seen as a weakness of our study, not allowing us to stratify this group according to tau pathology. However, Thorpe et al (2004) identified PIN1 depletion in FTD with and without tau pathology [7].

Additionally, we identified four novel *PIN1 *sequence variants in six patients with familial AD (Table 3). One variant was found in the *PIN1 *promoter region (g.9805834T>C), one in exon 1 (a silent substitution c.24C>T) and two in introns 1 and 3 (c.58+64C>T and c.382+105C>T). None of them was found in the previous studies [26,28]. Variant c.382+105C>T due to its location is rather unlikely to affect splicing of *PIN1*. The putative role of other variants is described below. All of them could potentially influence *PIN1 *expression and/or splicing.

### In silico analysis of g.9805834T>C variant

*In silico *analysis of the identified g.9805834T>C promoter variant using Matinspector predicted that it could disrupt the binding sites for four transcription factors/transcription factor families: FAST-1 SMAD interacting proteins, PAR/bZIP family, CCAAT/Enhancer Binding Protein (C/EBPs) and Ikaros zinc finger family. Similar analysis performed by another tool, Mapper, confirmed loss of the sites for C/EBPs and PAR/bZIP family, which could be due to similarities between the consensus sequences recognized by both leucine zipper transcription factor families. In both analyses C/EBP binding site obtained higher matrix similarity scores than the PAR/bZIP family.

The CCAAT/Enhancer Binding Proteins (C/EBPs) belong to the superfamily of transcription factors, which includes c-Jun, c-Fos and cAMP response element binding protein (CREB). C/EBPβ is required for neuronal differentiation, maturation and apoptosis [43-45]. Additionally, it plays an important role in the consolidation of mammalian long-term memory and in synaptic plasticity [46,47].

The activity of C/EBPs depends on the phosphorylation status of their Ser/Thr-Pro motifs and PIN1 was suggested to participate in their post-translational modifications [48]. Mutual interactions of both proteins can affect their common partner, E2F. It was demonstrated that *PIN1 *expression is mediated by E2F [49] and that C/EBPβ regulates E2F target gene activation by interacting with E2F and presumably by binding to their promoters [16]. Analysis of the whole *PIN1 *promoter region revealed one (Mapper) or two (Matinspector) binding sites for C/EBPs in the promoter sequence. Therefore, the loss of C/EBP binding site by g.9805834T>C variant might affect EF2 mediated activation of *PIN1 *transcription.

### In silico analysis of c.24G>T variant

c.24G>T is a synonymous substitution (8Pro, CCG>CCT), localized in a region participating in the PIN1 WW domain formation, responsible for binding hyperphosphorylated Tau [5]. ConSeq predicted that 8Pro is a highly conserved residue. In addition, Multiple Sequence Alignment revealed that this position is highly conserved between human PIN1 and its homologs in several species (e.g. *Pan troglodytes, Canis lupus familiaris, Bos taurus*, *Drosophila melanogaster*). Moreover, the proline is also conserved between human PIN1 and its yeast ortholog, Ess1p.

Despite c.24G>T variant does not affect protein coding, it might disrupt specific splicing elements. Recently, it has been acknowledged that silent changes have the potential to alter the efficiency and specificity of splicing, and contribute to phenotypic variability [50,51].

Analysis of exon 1 sequence by software that detects exonic splicing enhancer (ESE) sites indicated that c.24G>T variant is located within five putative ESE (Table 4). Importantly, using the default settings of ESEfinder, the program predicted that this transversion might disrupt one putative ESE recognized by SF2/ASF, reduce high score of another SF2/ASF motif and enhance binding of SF2/ASF (IgM-BRCA1). ESEfinder anticipated that c.24G>T mutation might shift a putative responsive site for SC35 four nucleotides downstream *PIN1 *sequence. Moreover, c.24G>T could increase binding of SRp55 and generate SRp40 motif. However, the new putative SRp40 motif (CCTCCCG) would overlap with the recognition site for SF2/ASF (IgM-BRCA1) (CTCCCGG). Simultaneous binding of two overlapping ESEs is considered as rather unlikely [51].

**Table 4:** Effect of c.24G>T mutation on calculated exonic splicing enhancer motifs scores.

SR protein	c.24G	c.24T
**SF2/ASF (1)**	Score = 2.02	**0**
**SF2/ASF (2)**	Score = 4.42	↓ Score = 3.75
**SF2/ASF (IgM-BRCA1)**	Score = 2.758	↑ Score = 4.13
**SC35 (1)**	Score = 2.93	**0**
**SC35 (2)**	0	↑ Score = 3.54
**SRp40**	**0**	Score = 2.88
**SRp55**	Score = 3.02	↑ Score = 3.83

Only motifs distinguishing wild type DNA sequence and the one with mutation c.24G>T, with scores above the thresholds, are presented.

### Analysis of c.58+64C>T variant

Variant c.58+64C>T (according to the GenBank accession numbers NM_006221 and NC_000019.8) was identified in three female patients, one with familial EOAD (fEOAD) and two with familial LOAD (fLOAD). The earlier age at onset of the patient with EOAD (54 years) could be explained by carrying two alleles of *APOE4 *in comparison with two LOAD patients (aged 70 and 71 years) with the same variant but genotype *APOE3/4*. As dose-effect relation data on *APOE4 *allele suggests, homozygosity for *APOE4 *might have accelerated the age at onset in carrier of c.58+64C>T variant [52].

Importantly, c.58+64C>T substitution is located in an alternative exon found selectively in testis (The AceView genes: http://www.ncbi.nlm.nih.gov/IEB/Research/Acembly). An online tool, Automated Splice Site Analysis, revealed that c.58+64C>T substitution increases 14.3 fold the strength of a potential binding site for SC35 situated one nucleotide upstream the variant. Previously, SC35 was reported to be responsible for aberrant splicing of the E1α Puryvate Dehydrogenase (PDHA1) mRNA in mental retardation with lactic acidosis [53,54]. Moreover, RegRNA program revealed that the c.58+64C>T substitution introduces two intronic splicing enhancers, an intronic AGGG motif (on + strand) and CTGC (on - strand). (A/U)GGG motif was shown to enhance alternative splicing of the chicken beta-tropomyosin pre-mRNA [55], thus introduction of another (A/U)GGG motif to the three preexisting ones in the 5' part of intron 1 could affect its splicing.

Variant c.58+64C>T in the patient with AAO of 51 years was associated with a 2.82 fold decreased *PIN1 *expression in the blood leukocytes. As decreased PIN1 level was previously observed in brains of Alzheimer's disease patients [2,7,15], it raises the possibility that identified variant could exert more profound effects in the brain.

Presented results concerning c.58+64C>T variant are consistent with the hypothesis linking *PIN1 *downregulation with amyloidogenic APP processing and aberrant cell cycle re-entry. As it stemmed from the observations of Fanghänel and coworkers (2006), even a basal level of PPIase activity complement a lethal cell cycle dysfunction related to PIN1 dysfunction at the single cell level [56]. However, in organs with high levels of PIN1 activity, like brain and testis, the basal activity is not sufficient to complement the pathology. This observation suggests that decrease in PIN1 activity in human brain could lead to neurodegeneration. The deleterious effect could be mediated either by oxidative stress, or by rare *PIN1 *sequence variation.

## Conclusion

Despite our data do not directly support the role of common PIN1 polymorphisms as AD risk factor, presented results concerning c.58+64C>T variant could be interpreted as being consistent with the hypothesis linking PIN1 downregulation with amyloidogenic APP processing and aberrant cell cycle re-entry. Presented data of the identification of four new *PIN1 *sequence variants underscore the importance of further studies on *PIN1 *variation in familial AD patients. None of the variants was connected with very early onset AD and/or rapid progression of the disease. Therefore, our findings suggest that *PIN1 *variants could be causally connected with familial AD with age at onset ranging from 45 to 75 years.

## Competing interests

The authors declare that they have no competing interests.

## Authors' contributions

AM and CZ conceived this study. AM and KS performed statistical analysis. AM performed *in silico *analysis. AM, KJ, MO, and MB performed described experiments

KG, BKH, Maria Styczyńska, JS, DC, AT, and MB collected blood, mRNA and clinical data.

AM, CZ, and KS participated in the management, analysis, interpretation of the data and drafting of the manuscript. All authors read and approved the manuscript.

## Pre-publication history

The pre-publication history for this paper can be accessed here:

http://www.biomedcentral.com/1471-2350/10/115/prepub

## Supplementary Material

Additional file 1 **Supplementary Table 1**. Primers used for *PIN1 *gene amplification. Click here for file

Additional file 2 **Supplementary Table 2**. DHPLC temperature for the analysis of *PIN1 *gene fragments. Click here for file

Additional file 3 **Supplementary Table 3**. Primers used for real-time PCR for *PIN1 *and *SDHA *are shown. Click here for file
