# Supplementary material for: PIN1 gene variants in Alzheimer's disease
Source: BMC Med Genet. 2009 Nov 12;10:115. doi: 10.1186/1471-2350-10-115 (PMC2781804; doi:10.1186/1471-2350-10-115)
Supplement: Additional file 1 — Supplementary Table 1. Primers used for PIN1 gene amplification. [file 1471-2350-10-115-S1.docx]

Symbol PROM indicates a promoter fragment. Exon 1 was amplified together with the proximal promoter fragment (PROM5+exon1). Exon 4 was amplified in two overlapping parts, thus two sets of primers were used.

| Primer name | Primer sequence (5’->3’) | annealing temperature (ºC) |
| --- | --- | --- |
| PROM1F | ATGAAGTCTCGCTCTGTCACCT | 60 |
| PROM1R | GCAGTAGAAGGCTAAGAGAAGTCTG | 60 |
| **PROM2F** | ATTGGGAAACCGAAGTACAGAGTA | 60 |
| **PROM2R** | TTGGAACCTGCTTACTTCCTTTATT | 60 |
| **PROM3F** | CCGTGTTCTTATATGAATGTCAATG | 60 |
| **PROM3R** | GGGATAGAGCTTATGGATTGGCTA | 60 |
| **PROM4F** | TTGCTATGGCAACAGCAGAG | 60 |
| **PROM4R** | ATTGGCTTTCTGGCCTTCTATT | 60 |
| [PROM5+ex1]F | CCGCCCAGCCTTTCTTCTAC | 64 |
| [PROM5+ex1]R | CTCAGCTTCCTCAGGCTTCC | 64 |
| exon2F | GATACACCATGGATTTGTTGAATG | 60 |
| exon2R | GTGAAGGTCAGGGTCAGGTC | 60 |
| exon3F | GTATGTGTGAGGAGAGGGGTTGT | 63 |
| exon3R | CCTCTGAAGCCTGAGGAAGG | 63 |
| exon4aF | GATGAGTGTGGACGAGTGTGAG | 63 |
| exon4aR | GAAGTCAATTCCTTAAGGGAGAATC | 63 |
| exon4bF | CCTGTCCATCCCCAGTTG | 65 |
| exon4bR | AGCTGGGACCCTTAGGATG | 65 |
