# Supplementary material for: PIN1 gene variants in Alzheimer's disease
Source: BMC Med Genet. 2009 Nov 12;10:115. doi: 10.1186/1471-2350-10-115 (PMC2781804; doi:10.1186/1471-2350-10-115)
Supplement: Additional file 2 — Supplementary Table 2. DHPLC temperature for the analysis of PIN1 gene fragments. [file 1471-2350-10-115-S2.docx]

| *PIN1* amplicon | Product length (bp) | Optimal temperature of DHPLC separation (ºC) |
| --- | --- | --- |
| PROM1 | 473 | 58 |
| PROM2 | 475 | 61.5 |
| PROM3 | 446 | 62 |
| PROM5+ex1 | 446 | 71 |
| Exon2 | 499 | 65.5 |
| Exon3 | 428 | 66 |

DHPLC analysis was performed using a Hewlett-Packard 1050/1100 chromatographic system consisting of quaternary gradient pump, HP G1322A Vacuum Degasser, Rheodyne 7125 manual injection valve with 20 µl PEEK loop, HP G1316A thermostatted column compartment and UV variable wavelength detector (VWD) set at 260 nm with standard flow cell. The system was modified by replacing stainless steel tubing with PEEK wherever possible. The mobile phase was preheated between the injector and the Helix DNA column (50 × 3.0 mm, CP28353, Varian).

The PCR products were denatured at 95°C for 5 min and then re-annealed by decreasing temperature from 95 to 45°C over a period of 50 min. The PCR product (5–10 µl) was injected onto the column and separated using a linear acetonitrile gradient. The column mobile phase consisted of a mixture of 0.1 M triethylammonium acetate pH 7.0 (TEAA) with 0.1 mM ethylene diaminetetraacetic acid (EDTA) with (buffer B) or without 25% (v/v) acetonitrile (buffer A). The flow rate was 0.5 ml/min. After equilibration with a concentration 5% below the start value, the linear gradient of buffer B content (9% increase over 3 min from injection time) was adjusted to set the elution time of homoduplex peak to between 3.8 and 4.3 minutes. Between sample analyses, the column was regenerated with 95% buffer B for 0.5 min. Equilibration with concentration of buffer B 5% below the start value lasted for 2 min so the total time between injections was 5.5 min. The temperature for heteroduplex analysis was primarily established using DNA Melt software as described by Jones et al. (1999), which is available at http://insertion.stanford.edu/melt.html. The final temperature for optimal resolution of the homoduplexes and heteroduplexes for each fragment was experimentally determined.

Jones AC, Austin J, Hansen N, Hoogendoorn B, Oefner PJ, Cheadle JP, O'Donovan MC: Optimal temperature selection for mutation detection by denaturing HPLC and comparison to single-stranded conformation polymorphism and heteroduplex analysis. *Clin Chem* 1999; 45:1133-1140.
