# Supplementary material for: PIN1 gene variants in Alzheimer's disease
Source: BMC Med Genet. 2009 Nov 12;10:115. doi: 10.1186/1471-2350-10-115 (PMC2781804; doi:10.1186/1471-2350-10-115)
Supplement: Additional file 3 — Supplementary Table 3. Primers used for real-time PCR for PIN1 and SDHA are shown. [file 1471-2350-10-115-S3.docx]

| Primer name | Primer sequence (5’->3’) |
| --- | --- |
| PIN1f | AGTCGGGAGAGGAGGACTTT |
| PIN1r | CAAACGAGGCGTCTTCAAAT |
| SDHAf | actggatactgagcagaaatggaat |
| SDHAr | cgaggttttcacttcactgttga |
